# Protection of Antarctic microbial communities – ‘out of sight, out of mind’

**DOI:** 10.3389/fmicb.2015.00151

**Published:** 2015-02-25

**Authors:** Kevin A. Hughes, Don A. Cowan, Annick Wilmotte

**Affiliations:** ^1^British Antarctic Survey, Natural Environment Research CouncilCambridge, UK; ^2^Centre for Microbial Ecology and Genomics, Department of Genetics, University of PretoriaPretoria, South Africa; ^3^Bacterial Physiology and Genetics, Centre for Protein Engineering, Department of Life Sciences, University of LiègeLiège, Belgium

**Keywords:** Antarctica, conservation, microbial diversity, human impact, inviolate area

## Abstract

Recent advances in molecular biology techniques have shown the presence of diverse microbial communities and endemic species in Antarctica. Endemic microbes may be a potential source of novel biotechnologically important compounds, including, for example, new antibiotics. Thus, the scientific and biotechnological value of Antarctic terrestrial microbial habitats can be compromised by human visitation to a greater extent than previously realized. The ever-increasing human footprint in Antarctica makes consideration of this topic more pressing, as the number of locations known to be pristine habitats, where increasingly sophisticated cutting-edge research techniques may be used to their full potential, declines. Examination of the Protected Areas system of the Antarctic Treaty shows that microbial habitats are generally poorly protected. No other continent on Earth is dominated to the same degree by microbial species, and real opportunities exist to develop new ways of conceptualizing and implementing conservation of microbial biogeography on a continental scale. Here we highlight potential threats both to the conservation of terrestrial microbial ecosystems, and to future scientific research requiring their study.

## INTRODUCTION

Antarctica is the most pristine and extreme continent, and as such, harbors a diversified and adapted biodiversity (Laws, 1984). Food chains are reduced in terrestrial biotopes with the permanent inhabitants predominantly restricted to microinvertebrates, cryptogams, and microorganisms. Ice-free ground in Antarctica only covers 44,000 km^2^ (or 0.34% of the continent’s 14 million km^2^ area) but hosts the bulk of terrestrial Antarctic life (Convey et al., 2012). Microorganisms, including bacteria, algae, fungi, and viruses, generally comprise the majority of the biomass and biodiversity in Antarctic terrestrial and freshwater ecosystems, particularly under more climatically extreme habitats where higher organisms cannot survive (Friedmann, 1993; Cowan et al., 2010a). Terrestrial sites may contain microbial communities that have persisted in Antarctica for at least several glacial cycles (Convey et al., 2008; De Wever et al., 2009), or species that have colonized more recently by natural processes such as wind currents or transport by migrating birds (Schlichting et al., 1978; Pearce et al., 2009).

In recent years, many important insights into Antarctic microbial ecology have been achieved using molecular biological techniques, including that microbial biodiversity differs at sites across Antarctica and high levels of endemism exist in some microbial groups, despite the widely assumed ease of natural colonization processes (Vincent, 2000; Lawley et al., 2004; De Wever et al., 2009; Cowan et al., 2010a; Vyverman et al., 2010; Peeters et al., 2011). Furthermore, microorganisms can exist in complex assemblages and at higher levels of diversity than estimated by earlier studies based upon microscopic and culture-dependent techniques and Antarctic microorganisms have specialized genes that enable them to survive and function in inhospitable polar habitats (D’Amico et al., 2006; Yergeau et al., 2007, 2009; Sawstrom et al., 2008; López-Bueno et al., 2009; Pointing et al., 2009; Cary et al., 2010; Casanueva et al., 2010; Chong et al., 2012). In recent years, molecular biological techniques have dramatically increased in sophistication and reduced in cost. As these trends continue, understanding of scientific issues of local and global relevance, such as the effects of climate change on microbial systems, are likely to advance rapidly (Wall, 2007; Loman et al., 2012). Furthermore, communities in pristine habitats will potentially show discrete patterns of adaptation and evolution, including novel physiologies and biochemical processes, which could inform our understanding of microbial ecology. In this context, it is important to ask what threats microbial communities face and whether adequate protection is given to this unique and valuable scientific resource.

## ANTHROPOGENIC CONTAMINATION OF MICROBIAL TERRESTRIAL HABITATS

Human activity within Antarctic has increased greatly since the 1950s, initially due to nations’ growing geo-political and scientific interests in the region and the subsequent establishment of research stations and other infrastructure, followed by the rapid expansion of the tourism industry since the start of the new millennium. Despite the continent’s vast size, ice-free habitats available for terrestrial colonization are scarce with only c. 6000 km^2^ of ice-free ground found within 5 km of the coast. This area, which accommodates the majority of the more highly developed terrestrial communities as well as the haul out and breeding sites of Antarctic marine mammals and avifauna, is increasingly impacted by human activities, such as construction of research stations, roads, airstrips, and other infrastructure with the associated pollution and habitat disruption, and destruction (Tin et al., 2009; Braun et al., 2014). Such activities have been identified as a major threat to Antarctic ecosystems and the on-going expansion of human footprint in the region will inevitably impact upon more remote and potentially more vulnerable biological communities (Hughes et al., 2011; Chown et al., 2012; Convey et al., 2012).

In Antarctic terrestrial sites, the introduction of invasive non-native plants and animals or chemical pollution can be highly visible, with a clear impact. In contrast, microbial or genetic contamination is not visually obvious and is not considered routinely by non-microbiologists, never mind those undertaking compulsory Environmental Impact Assessments, as required by the Protocol on Environmental Protection to the Antarctic Treaty. However, long-term impacts on microbial biodiversity can occur. Non-native microorganisms introduced from areas outside Antarctica, or from other biologically-distinct areas of Antarctica, may possess ecological, biochemical, or physiological capabilities that alter the structure and function of the indigenous community. The introduction of invasive non-native microbes could potentially lead to a loss of native microbial biodiversity. Added to this, non-native species may contain genes that encode properties not found normally in pristine Antarctic microbial communities (e.g., resistance to antimicrobial agents or other physiological or biochemical benefits). These genes may be transferred from the non-native species to indigenous microorganisms by lateral gene transfer. If the genes are expressed in indigenous microorganisms, they may alter the functional properties of the individual species and the community as a whole (Baker et al., 2003; Storrie-Lombardi and Williamson, 2010; Cowan et al., 2011a).

Introduced non-native microorganisms and microbial genetic contamination have been identified in association with dust, equipment, and fibers from clothing (Broady and Smith, 1994; Shukla et al., 1999; Cowan and Ah Tow, 2004; Takashima et al., 2004; Teufel et al., 2009), human waste (Sjöling and Cowan, 2000), and shed human skin cells. Indeed, it is estimated that a person may shed c. 300 million dead skin cells per day of which c. 10% may carry commensal microorganisms (Wilson, 2001; Cowan et al., 2011a). Therefore, visits to Antarctic terrestrial sites by small numbers of people, or even individuals, will inevitably result in the release of some microbial contaminants. Culture-dependent and molecular studies have already shown that microbial contamination resulting from human activities can be detected in Antarctic soils long after the activities have ceased (Hirsch et al., 1985; Nedwell et al., 1994; Sjöling and Cowan, 2000; Baker et al., 2003; Hughes and Nobbs, 2004; Cowan et al., 2011a).

## DO WE KNOW WHICH LOCATIONS ARE FREE OF POTENTIAL MICROBIAL CONTAMINATION?

The human footprint in Antarctica continues to expand, but our records and understanding of which areas have been visited is not yet comprehensive. Furthermore, there may be limited temporal and spatial precision in locating past scientific activities, and such efforts become more difficult with passing time and increasing human activity. Therefore, it is increasingly difficult to identify pristine areas that are known categorically to be free of earlier human activity, and that could represent the most appropriate locations for microbiological research using sophisticated technologies where minimal prior contamination is an essential requirement (Hughes et al., 2011). Undertaking general scientific activities at a site may compromise, to some degree, the value of that location for future molecular biological research. Given the technological advances in molecular biological methods over the past decade, we may reasonably expect that future scientists will have access to technologies with vastly greater scope and sensitivity than those we currently use. A better understanding of which areas remain free of all past human activity may help inform conservation planning for microbiological habitats. Such locations may include inland sites situated far from research stations and logistics hubs.

It is also possible that human activities at one site may compromise the pristine nature of geographically discrete sites. Little is known of the extent of local (rather than regional or global) aeolian transport of particles and survival and persistence of their associated microorganisms. Data from a few aerobiological studies have shown the presence of viable microorganisms and of their genomic material in the atmosphere above Antarctic localities (reviewed by Pearce et al., 2009). The aerial transport of microorganisms has been shown at different scales, from the global long-distance travel in the high atmosphere to local near-ground aeolian redistribution (Pearce et al., 2009, 2010; Sabacka et al., 2012). However, transport is only the first requirement for dissemination and must be followed by successful colonization of new habitats. Therefore, studies of microbial biodiversity patterns in Antarctic biotopes were recently conducted to detect the signature of dispersal mechanisms in these patterns (Sokol et al., 2013; Herbold et al., 2014). Indeed, the microbial communities appeared to be influenced, not only by the environmental characteristics of their biotopes, but by dispersal dynamics and they showed particular spatial distributions.

## HOW WELL DO WE PROTECT MICROBIAL HABITATS WITHIN ANTARCTICA?

For conservation purposes, the small scale of microorganisms is a real handicap that hinders the appreciation of their uniqueness, role, and their risk of extinction (Griffiths, 2012). ‘Extinction’ is a concept from which microorganisms have generally been excluded. Higher species, such as penguins and seals, look ‘cute’ or move in a charismatic manner, creating an emotional attachment with the public for these iconic animals and inevitably biasing conservation efforts towards protection of these species. In addition, the more complex methods needed to identify microorganisms have had the effect of setting them aside or exclude them from general environmental biodiversity studies largely based on the visible characteristics of organisms (Malaterre, 2013). The absence of a visual link with microorganisms and a lack of understanding of their importance within ecosystems by the public (and, in some cases, policy-makers) make the protection of the microbial habitats in Antarctica more difficult to advocate. The challenge for those interested in microbial conservation will be communication to the general public of the importance of microbial species.

The Antarctic Treaty Consultative Meeting (ATCM) is the forum for all decision-making within the Antarctic Treaty area, which encompasses all land, sea, and ice shelves south of latitude 60°S. The Committee for environmental protection (CEP, 2011) makes recommendations to the ATCM on the implementation of the Protocol on Environmental Protection to the Antarctic Treaty (also known as the Madrid Protocol or Environmental Protocol), the overarching aim of which is the protection of Antarctica as ‘a natural reserve devoted to peace and science’. A key feature of the Protocol is that it prohibits collection of mineral resources for purposes other than scientific investigation, in effect banning mining activities. The Protocol has an indefinite duration, but Article 25 does provide for a review process 50 years after the Protocol entered into force (i.e., 2048). Modifications may then be made, but only with the agreement of a majority of the nations that were Antarctic Treaty Consultative Parties when the Protocol was adopted in 1991 (i.e., 26 Parties). Should future large scale mining activities be undertaken, it is likely that they would put existing marine and, in particular, terrestrial habitats under substantial threat of destruction or contamination. This makes the use now of existing legislation and established management tools that allow protection of species and habitats all the more important.

The Protocol has five Annexes in force, each of which deals with a different environmental issue. The very fact that Annex II ‘Conservation of Antarctic fauna and flora’ lacks any reference to lower taxonomic groups in its title gives an indication of how little microorganisms and their dominance in Antarctic ecosystems were understood by the authors and how little they are considered in the rest of the Annex. Rather than highlighting the importance and uniqueness of microbial species and setting out their protection, the Annex considers them only in a negative manner, for example, to prevent their introduction with food, soil, or in the context of experiments.

Annex V to the Protocol concerns Areas Protection. Since coming into existence in 1998, the CEP has had responsibility for the network of Antarctic Specially Protected Areas (ASPAs) that has evolved in an unsystematic manner over the past 50 years. The ASPAs are intended to protect “outstanding environmental, scientific, historic, esthetic, or wilderness values, any combination of those values, or ongoing or planned scientific research” (http://www.ats.aq/e/ep_protected.htm); however, the level of success for most of these values is in question, not least the environmental values (Shaw et al., 2014). A closer look at the level of protection of microorganism and microbial habitat within the Protected Areas system shows little protection, compared with the vast scale of the continent (http://www.ats.aq/e/ep_protected.htm). Of the 72 ASPAs that exist currently, ca. 55 protect terrestrial habitats, and predominantly vascular plant and bryophyte communities, in a combined area of less than 700 km^2^ (Shaw et al., 2014). Lichens (symbionts of fungi and algae and which constitute a major element of the Antarctica’s macroscopic terrestrial biota) are included in the description of protected values in 28 ASPAs. However, other microbial groups are less-well represented with algae protected in 16 ASPAs, cyanobacteria in 7 and snow algae in 3. Eight ASPAs mention protection of ‘microbial habitats’ or ‘microbial communities’ or ‘soil and lake microflora’ but with little mention of specific bacteria, fungi, or viruses, indicating little understanding of their true value in these systems. In many cases, microorganisms are protected, apparently almost as an afterthought, as a secondary value within ASPA management plans. Nevertheless, some protection of specific microbial communities is found in the protected area system. Within the McMurdo Dry Valley, cryptoendolithic microbial communities are protected within ASPA 138 Linneaus Terrace, while ASPA 172, that encompasses the lower Taylor Glacier including Blood Falls, protects a visually striking microbial community of apparently marine origin. Also in southern Victoria Land, ASPA 175 safeguards the biodiversity in microbial-dominated high altitude geothermally heated areas on volcanoes Mount Erebus, Mount Melbourne, and Mount Rittmann, while in the remote Pensacola Mountains, ASPA 119 protects cyanobacteria-dominated terrestrial and freshwater ecosystems. All four locations share a rare characteristic: the general absence of higher biological groups, which allows the extraordinariness of the resident microbiological communities to shine through. However, protection of microbial communities – as the dominant value worthy of protection – in more complex biological communities, where their presence is less obvious, is almost absent from the Antarctic Protected Areas system.

An excellent model for future microbial community protection, based on the Linnaeus Terrace precedent, actually exists, albeit not formally considered. The low altitude maritime valley complex comprising the Miers, Marshall, Garwood, and Shangri La Valleys is rich in hypolithic microbial communities (localized and specialized microbial communities on the ventral surfaces of translucent quartz pebbles). No other low altitude valley has the same number or diversity of the communities, which are largely or completely absent in the high altitude valleys. Given the specialized nature of these communities, their acknowledged role as biodiversity hotspots and as key sites for N and C cycling, there is every justification for considering protected status for this region (Cowan, 2009; Cowan et al., 2010a, 2011b; Khan et al., 2011; Chan et al., 2012; Gokul et al., 2013; Makhalanyane et al., 2013; De Maayer et al., 2014).

## A WAY FORWARD

Studies focusing predominantly on Antarctica’s macroscopic biota, which acknowledge that biodiversity information is lacking for many Antarctic eco-regions, have concluded that the current protected areas system is inadequate and does not protect a representative selection of Antarctica’s biodiversity and range of habitats (Terauds et al., 2012; Shaw et al., 2014). In comparison, microbial biodiversity data across Antarctica is much less well known, which creates substantial challenges for scientists, conservation professionals, and policy-makers (Cowan and Ah Tow, 2004; Cowan et al., 2010b). As a first step to help solving these questions, an extension of the database www.biodiversity.aq is presently developed to give access to microbial diversity (meta)data, i.e., the Microbial Antarctic Resources System (mARS; Murray et al., 2013). As one of, if not the most important biological group in Antarctica, due to their presence under extreme polar condition and importance for ecosystem function, microbial protection should be in the forefront of policy-makers minds when considering Antarctic conservation.

It will be up to microbiologists working in Antarctic to provide robust evidence and push for this protection, both in relation to areas they know and work in themselves, but also as a more general concept so that as yet little visited areas of Antarctica will have their microbiological values considered and protected too. During field expeditions, the possibility of aerial dispersal of microorganisms from local, and maybe regional sources, should be taken into account to protect areas that could be affected (situated down-wind…). In addition, a concerted international effort to study the microbial diversity in the atmosphere above the whole Antarctic continent would be needed to estimate the influx of new microorganisms to terrestrial communities and their impact on those.

Scientist from many countries working within the Scientific Committee on Antarctic Research (SCAR) programmes AnT-ERA (Antarctic Thresholds – Ecosystem Resilience and Adaptation) and AntEco (State of the Antarctic Ecosystem) may be well placed to take up this challenge. We currently do not have adequate protection of representative habitats across Antarctica, where microbial research techniques (including the need for biosecurity standards of the highest levels) are taken into consideration in the writing of the management plan. This has been done to some degree in ASPA 126 Byers Peninsula, where Restricted Zones have been created to protect areas of microbiological interest, but management tools such as these are little used elsewhere in Antarctica. Fortunately, some progress is being made with international discussion commencing on how the microbiological values present within Antarctica’s rare areas of geo-thermally heated ground might be protected (New Zealand et al., 2014). However, perhaps it is time for the Antarctic Treaty Parties to set aside concerns over universal access within Antarctica (as stipulated in Article 7) and consider the designation of truly ‘inviolate’ areas that can be set aside for future research in 20, 50, or even 100 years time (Hughes et al., 2013). By then, these locations may be the last areas of Earth known to have remained unvisited, and therefore be of immense scientific worth. Inevitably, management protocols and quarantine measures may place an additional burden on researchers of other scientific disciplines (e.g., botany or geology) who want to work in a restricted area. However, with adequate communication and understanding, there is much scope for compromise, leading to protection of both Antarctic microbial habitat, and future science opportunities. Given that protection of endemic microbial diversity in other areas of the Earth is generally little considered, never mind put in to practice, the Antarctic community has an opportunity to lead the development of broad-scale policies and management actions to conserve microbial diversity, some of which may be applicable globally.

Much effort has been devoted to developing cutting-edge technologies and guidelines to facilitate access to sub-glacial lakes, while at the same time conserving and protecting these pristine environments (Scientific Committee on Antarctic Research [SCAR], 2011), even though the effectiveness of the methodologies used at some locations has been questioned (Schiermeier, 2013). However, clean technologies to allow sampling of pristine Antarctic terrestrial environments to equivalent high standards have not been developed. Furthermore, in practical terms, we still have no convenient robust cold weather clothing systems capable of delivering the high levels of biosecurity or quarantine needed to keep sites microbiologically pristine (Lankester et al., 2002; Owers et al., 2004; Scientific Committee on Antarctic Research [SCAR], 2009; Committee for environmental protection [CEP], 2011; Eisen, 2011; Hickman-Davis et al., 2012). There is still a great deal we do not know about the rate and scale of contamination of microbial habitats, such as the scale and kinetics of transport processes for non-native microorganisms after the initial introduction event or how microbial diversity and function are altered following human visitation (Miwa, 1976; Toyoda et al., 1985; Abyzov et al., 1986; Kerry, 1990; Ah Tow and Cowan, 2005; Pearce et al., 2010) Further research into these areas may help safeguard the pristine nature of some terrestrial microbial habitats.

Let us hope that in the near future Antarctica’s pristine microbial habitats may be recognized for their scientific value, for the realization that they may also have an economic value is growing. Bioprospecting activities continue to occur within the Treaty area with genetic and biochemical resources being increasing recognized and exploited (Hemmings and Rogan-Finnemore, 2008). This has created tensions within the Treaty system regarding benefit sharing, but more critically puts microbiologist in the roles of both potential exploiters of and conservation champions for Antarctic microbial biodiversity (Hemmings, 2010). As a consequence, managing closely inter-related scientific and commercial biotechnological activities in Antarctica could create both challenges and opportunities for those seeking to protect Antarctica’s microbial habitats. Either way, Treaty Parties would be well-advised to take the short and longer term conservation of Antarctic microbial resources seriously, before their conservation and commercial values are compromised irreversibly.

## Conflict of Interest Statement

The authors declare that the research was conducted in the absence of any commercial or financial relationships that could be construed as a potential conflict of interest.
